# Radiomics-driven spectral profiling of six kidney stone types with monoenergetic CT reconstructions in photon-counting CT

**DOI:** 10.1007/s00330-024-11262-w

**Published:** 2024-12-12

**Authors:** Alexander Hertel, Matthias F. Froelich, Daniel Overhoff, Tim Nestler, Sebastian Faby, Markus Jürgens, Bernhard Schmidt, Abhinay Vellala, Albrecht Hesse, Dominik Nörenberg, Rico Stoll, Hans Schmelz, Stefan O. Schoenberg, Stephan Waldeck

**Affiliations:** 1 Department of Radiology and Nuclear Medicine, University Medical Center Mannheim, University of Heidelberg, Mannheim, Germany; 2 Department of Diagnostic and Interventional Radiology, Federal Armed Services Hospital Koblenz, Koblenz, Germany; 3 Department of Urology, Federal Armed Services Hospital Koblenz, Koblenz, Germany; 4 Department of Urology, University Hospital Cologne, Cologne, Germany; 5 Siemens Healthcare GmbH, Forchheim, Germany; 6 Urinary Stone Analysis Center Bonn, Bonn, Germany

**Keywords:** Kidney stones, Radiomics, Photon-counting CT, Spectral profiling, Machine learning

## Abstract

**Objectives:**

Urolithiasis, a common and painful urological condition, is influenced by factors such as lifestyle, genetics, and medication. Differentiating between different types of kidney stones is crucial for personalized therapy. The purpose of this study is to investigate the use of photon-counting computed tomography (PCCT) in combination with radiomics and machine learning to develop a method for automated and detailed characterization of kidney stones. This approach aims to enhance the accuracy and detail of stone classification beyond what is achievable with conventional computed tomography (CT) and dual-energy CT (DECT).

**Materials and methods:**

In this ex vivo study, 135 kidney stones were first classified using infrared spectroscopy. All stones were then scanned in a PCCT embedded in a phantom. Various monoenergetic reconstructions were generated, and radiomics features were extracted. Statistical analysis was performed using Random Forest (RF) classifiers for both individual reconstructions and a combined model.

**Results:**

The combined model, using radiomics features from all monoenergetic reconstructions, significantly outperformed individual reconstructions and SPP parameters, with an AUC of 0.95 and test accuracy of 0.81 for differentiating all six stone types. Feature importance analysis identified key parameters, including NGTDM_Strength and wavelet-LLH_firstorder_Variance.

**Conclusion:**

This ex vivo study demonstrates that radiomics-driven PCCT analysis can improve differentiation between kidney stone subtypes. The combined model outperformed individual monoenergetic levels, highlighting the potential of spectral profiling in PCCT to optimize treatment through image-based strategies.

**Key Points:**

***Question*** *How can photon-counting computed tomography (PCCT) combined with radiomics improve the differentiation of kidney stone types beyond conventional CT and dual-energy CT, enhancing personalized therapy?*

***Findings*** *Our ex vivo study demonstrates that a combined spectral-driven radiomics model achieved 95% AUC and 81% test accuracy in differentiating six kidney stone types.*

***Clinical relevance*** *Implementing PCCT-based spectral-driven radiomics allows for precise non-invasive differentiation of kidney stone types, leading to improved diagnostic accuracy and more personalized, effective treatment strategies, potentially reducing the need for invasive procedures and recurrence.*

**Graphical Abstract:**

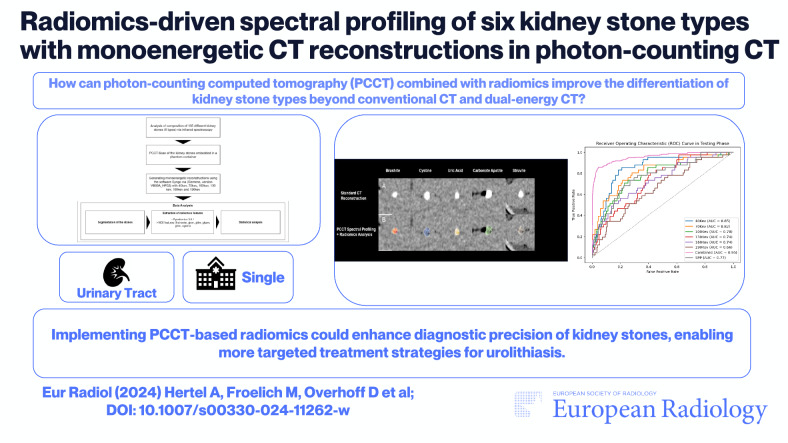

## Introduction

Urolithiasis is a common disease of the urinary tract associated with colicky pain and often accompanied by ureteral obstruction and consecutive urinary retention of the affected side [1]. Young as well as elderly people are affected with a broad etiological range, e.g., dietary habits, as well as genetic predispositions and other metabolic diseases and various medications, for example, antiviral agents [2, 3].

The gold standard for diagnosis is low-dose, non-contrast-enhanced computed tomography (CT) imaging of the abdomen [4]. Depending on the composition, size, and location of the stones, there are different therapeutic approaches. For this purpose, conservative therapy methods such as adequate pain therapy, an increase in the amount of drinking and physical exercise as well as drug therapy (for example, with alpha-blockers) to facilitate spontaneous discharge are used [5]. In the case of larger stones that do not drain spontaneously, invasive procedures such as ureterorenoscopy, percutaneous nephrolithotomy and extracorporeal shock wave lithotripsy are employed [4, 6, 7]. In patients prone to frequent stone formation, various methods of prophylaxis may be considered, including an increase in daily drinking, adjustment of eating habits, and possibly specific urinary stone metaphylaxis. Pharmacological chemolitholysis of urolithiasis can be indicated in select cases where the composition and size of the calculi suggest they are amenable to chemical dissolution. Different types of stones, such as uric acid stones or struvite stones, can potentially be dissolved by drug therapy [8]. Pharmacological chemolitholysis is a tailored approach, contingent upon a comprehensive evaluation of stone composition, patient health status, and the likelihood of stone dissolution with medical management [9–14].

To specifically adjust the prophylaxis or therapy, it is essential to know the chemical composition of the calculi. In particular, calcium oxalate stones, calcium phosphate stones, uric acid stones, struvite stones, and cystine stones are common.

There have been previous studies on CT-based composition analysis of kidney stones in regard to utilizing dual-energy computed tomography (DECT) and low-dose CT examinations [13, 15, 16].

Also, kidney stones and their compositions have already been studied in several preliminary works in CT examinations and the subsequently extracted radiomics features [10, 17].

Radiomics is a rapidly advancing field in medical image analysis, which goes beyond the limitations of human visual examination by delving into pixel-level data within medical images [18]. Its primary focus is on quantifying various metrics within a region or volume of interest, such as texture and shape features. This approach offers a potential expansion of the capabilities of medical imaging. With a substantial database, these features become extractable, suggesting the possibility of identifying novel characteristics that could serve as markers for specific pathologies or characteristics of different structures. Radiomics features have the potential to revolutionize medical imaging by serving as reliable biomarkers. After gaining initial traction in the realm of oncology, these features are now carving out a significant role across diverse research areas, including uroradiology, where they show great promise for enhancing acute diagnostic processes like those used in urolithiasis management [19–22]. Nonetheless, the uptake of radiomics in clinical practice is curtailed by challenges related to reproducibility and comparability of imaging features [23–25]. In the specific context of urological disorders, the diagnostic acuity for conditions such as urinary stone disease could be markedly improved by adopting these advanced imaging biomarkers. The advent of Photon-counting computed tomography (PCCT) has been a game-changer in this regard, offering significantly improved feature stability—a pivotal factor for the reliable differentiation of stone types—thus setting the stage for the integration of radiomics into routine clinical workflow [26].

Unlike conventional CT scanners that rely on energy-integrating detectors, PCCT employs cutting-edge photon-counting detectors capable of measuring individual x-ray photons. By differentiating between various energy levels of x-ray photons, it facilitates superior tissue contrast and mitigates image artifacts, thereby improving diagnostic accuracy.

The advent of PCCT marks a significant advance in medical imaging, particularly through its capacity to reduce radiation exposure while enhancing image quality via the discrimination of different energy spectra. This technology unlocks new avenues for multi-energy imaging and material differentiation within the body, crucial for the precise analysis and characterization of urinary stones [27–31]. Our ex vivo study aims to investigate whether photon-counting CT in combination with radiomics can provide a more accurate and detailed distinction between different types of urinary stones, with a particular focus on differentiating non-uric stone types. This approach seeks to support the development of non-invasive, personalized therapeutic strategies for patients.

## Materials and methods

### Study design

**Fig. 1:**
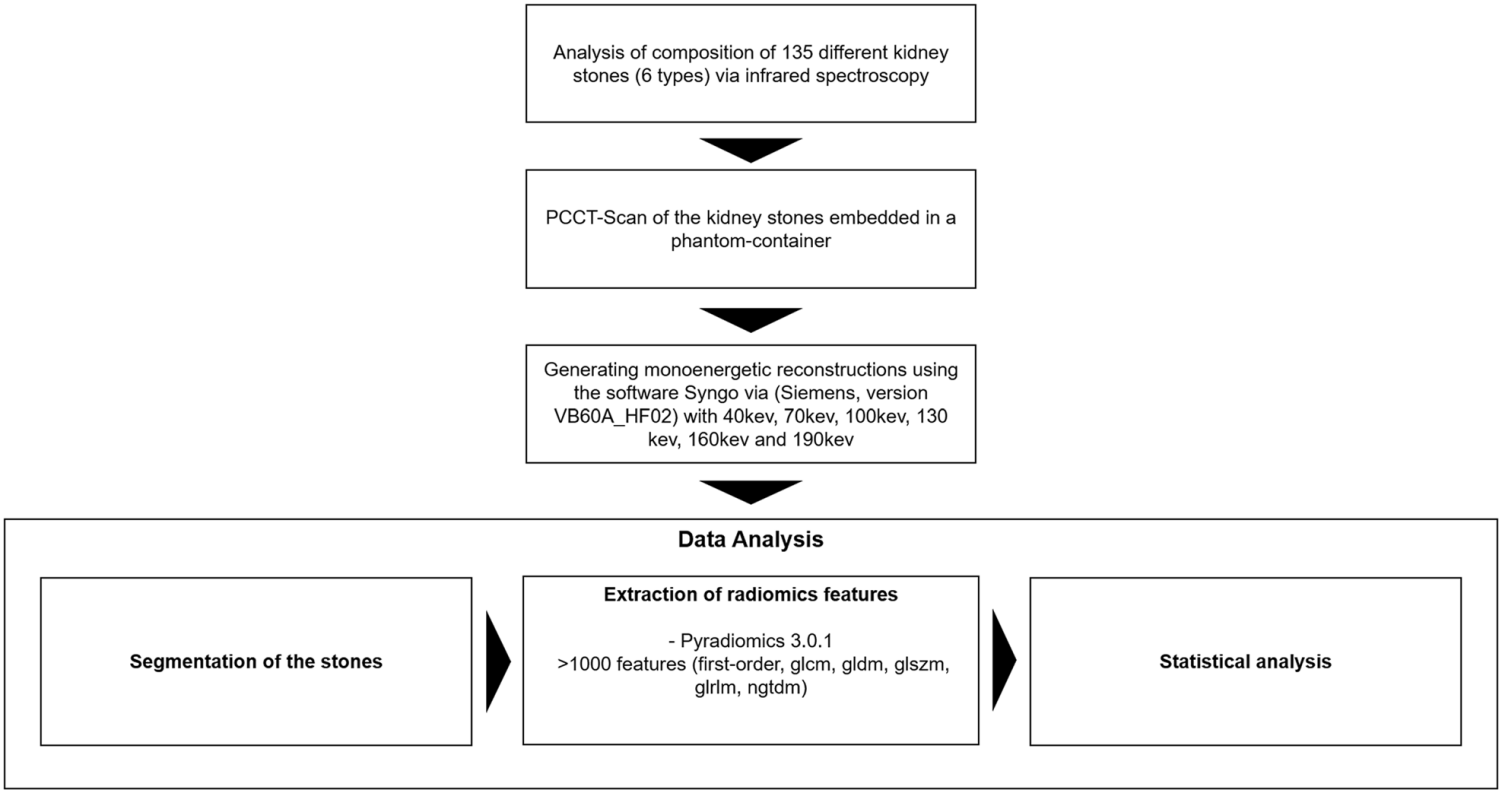
Flowchart of the established workflow

For this phantom-based single-center study, a total of 135 kidney stones were included, consisting of xanthine, brushite, carbonate apatite, cystine, struvite and uric acid stones. The stone types and their composition were examined in advance using infrared spectroscopy (Paragon 1000PC, PerkinElmer) at the German Urinary Stone Center in Bonn and established as a ground truth. The elaborated workflow of this study is presented in Fig. 1. The stones were measured manually using a digital caliper. The dataset has been analyzed regarding automated stone detection/analysis and volumetric/metric measurements and the results have been published in previous studies [32, 33].

### Phantom

**Fig. 2:**
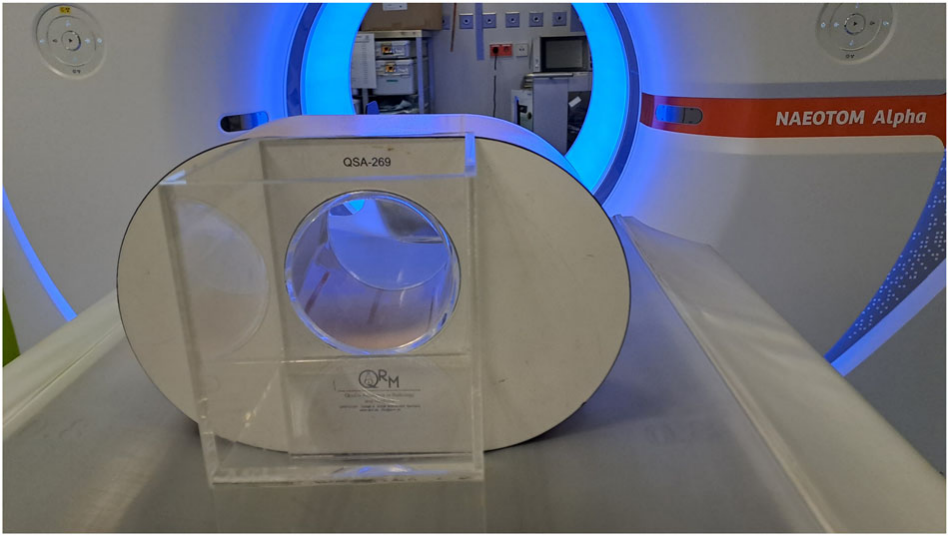
Image of the phantom

Ex vivo CT examinations were conducted at the Bundeswehrzentralkrankenhaus in Koblenz utilizing an abdomen phantom (QSA-269, produced in 2011 by QRM GmbH). This phantom is suitably employed as an anthropomorphic model for exploring the effects of scanning parameters in computed tomography. The materials comprising this anthropomorphic phantom are designed to replicate the x-ray attenuation properties of the human abdomen, being equivalent to soft tissue with an approximate value of 35 Hounsfield Units (HU) at 120 kV. The phantom features a standard borehole with a diameter of 100 mm, and its specifications include a total weight of 3.2 kg, with dimensions of 200 × 300 mm and a height of 100 mm. Figure 2 shows an image of the phantom.

For each scanning procedure, five urinary calculi were encased in a segment of red meat. This assembly was then shrink-wrapped, vacuum-sealed, and positioned centrally within the abdomen phantom, subsequently being encircled by water to closely simulate the surrounding human anatomy.

### PCCT imaging

The stones were scanned ex vivo using a first-generation dual-source photon-counting CT scanner (NAEOTOM Alpha, VA40, Siemens Healthineers). For PCCT imaging, the following parameters were employed: a tube voltage of 120 kV, a collimation of 140 × 0.4 mm, a rotation time of 0.5 s, and a spiral acquisition mode with a pitch of 0.8. The images were reconstructed utilizing a Qr40 kernel with a slice thickness of 1 mm. The field of view was 359 × 359 mm², the matrix 512 × 512. The increment was reconstructed with 0.7 mm.

### PCCT image analysis

Axial images of all scans were reconstructed with a slice thickness of 1 mm (increment 1 mm) using a soft tissue kernel (Qr40). The data was exported and stored in digital imaging and communication in medicine (DICOM) file format. Monoenergetic reconstructions of the PCCT scans were created using the Syngo.via software (version VB60A_HF02) from Siemens in 30 keV steps from 40 to 190 keV. Segmentation for each Urinary Stone was done semi-automatically by a clinical radiologist with 3 years of experience in segmentation, and subsequently, the segmentation and image data were converted to Neuroimaging Informatics Technology Initiative (NIFTI) file format for further processing with a dedicated segmentation tool (MITK workbench, Version 2021.10) [34].

### Radiomics feature extraction

The radiomics features of the prepared segmentations were extracted using a dedicated imaging biomarker standardization initiative definition-based python package (pyradiomics, version 3.0.1.) integrated in a custom-built docker container (Docker Desktop; Version 4.3.1, Docker, Inc.) [35].

Radiomics features were extracted in accordance with the PyRadiomics standard, including first-order, second-order (GLCM, GLDM, GLSZM, GLRLM, and NGTDM), shape features, as well as higher-order features such as wavelet and Laplacian of Gaussian (LoG) filters. The extraction was performed with voxel normalization, resampling to 2 × 2 × 2 mm and rebinning with fixed bin width of 25 HU. The Chebyshev distance is 1.

### Statistical analysis

Cluster Analyses and Box Plots were performed using R-Statistics (Version 2023.03.0+386) to explore the potential of radiomics for differentiating between different types of kidney stones. In addition, a Python-based RF classifier was applied. The classifier was trained with extracted radiomics parameters from each monoenergetic level at a time, the Spectral Post-Processing (SPP) datasets as well as in a combined, spectrally driven model approach with parameters from all monoenergetic reconstructions combined. For each trained classifier, the corresponding dataset was previously split into a training and test/validation group. To optimize its performance, a systematic hyperparameter tuning approach was adopted using grid search cross-validation. This technique involves exhaustively searching through a specified hyperparameter grid and evaluating each combination using cross-validation to identify the set of hyperparameters that yield the best performance. By leveraging this method, we aimed to enhance the model’s predictive accuracy and generalization ability, ensuring robustness in classification tasks.

Further, important features are extracted to understand the contribution of important radiomics features to classify the different types of kidney stones more effectively. Random forest calculates the difference between the prediction value at the leaf node and the prediction values at the nodes that precede it to get the estimated contribution of each feature.

## Results

**Fig. 3:**
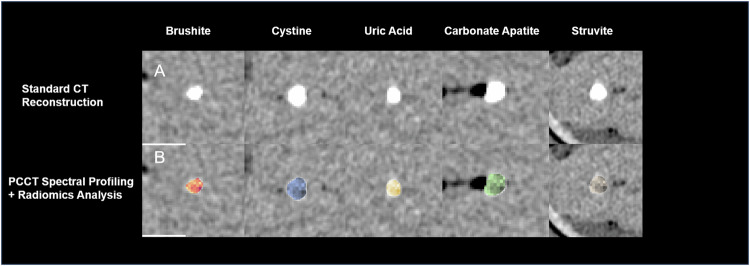
Photon-counting computed tomography scans of different kidney stone types and their spectral profiling & radiomics analysis. **A** The PCCT scan of various urinary stone types in a phantom setup showcasing the technology’s capability for high-resolution imaging. **B** The application of radiomics feature extraction and machine learning algorithms, highlighting the differentiation between the different kidney stone types

A total of 135 kidney stones were included in the final analysis. The examined kidney stones had an average volume of 0.15 mL. The longest diameter was 6.46 mm on average, the shortest diameter was 3.52 mm on average. The urinary stones examined consisted of 19 brushite stones (14.1%), 34 cystine stones (25.2%), 31 uric acid stones (23%), 20 carbonate apatite stones (14.8%), 17 struvite stones (12.6%) and 14 xanthine stones (10.3%) (Table 1). The stones were placed in the phantom enclosure described above and scanned in the PCCT. The average radiation dose for the scans of the phantoms was 0.84 mGy (CDTIvol) and 22.56 mGycm (DLP). The stones were then segmented semi-automatically, and all radiomics parameters were extracted from the different monoenergetic reconstructions. Figure 3 shows the scan result and illustrates the combined potential of radiomics analysis and spectral profiling for the differentiation of different kidney stone types.

**Table 1:** Descriptive statistics of the analyzed kidney stones

Number of stones	*n*
Total	135 (100%)
Brushite	19 (14.1%)
Cystine	34 (25.2%)
Uric acid	31 (23%)
Carbonate apatite	20 (14.8%)
Struvite	17 (12.6%)
Xanthine	14 (10.3%)

Average volume	mL
Total	0.15
Brushite	0.11
Cystine	0.49
Uric acid	0.04
Carbonate apatite	0.09
Struvite	0.23
Xanthine	0.11

Longest diameter	mm
Total	6.26
Brushite	5.08
Cystine	7.75
Uric acid	5.09
Carbonate apatite	6.59
Struvite	7.52
Xanthine	6.98

**Fig. 4:**
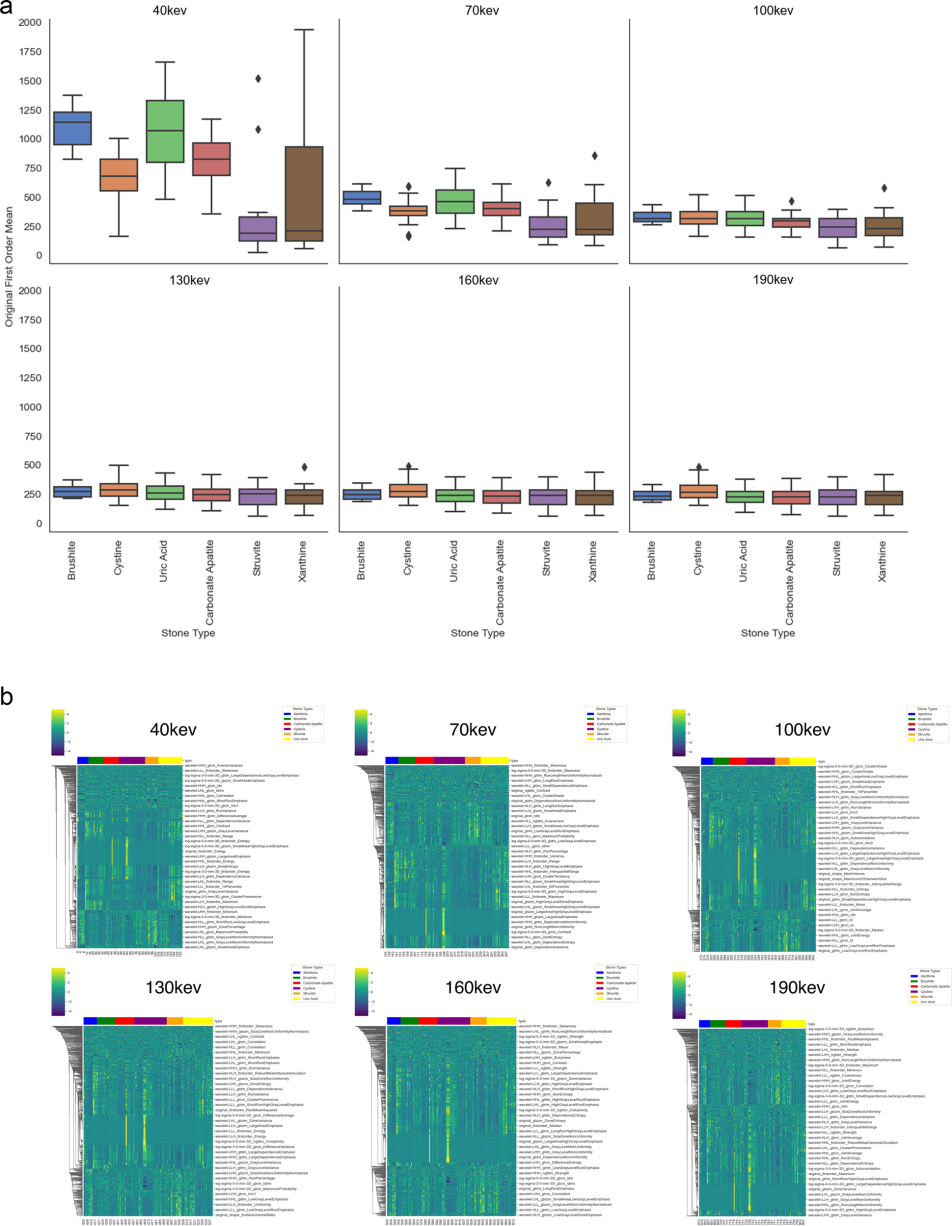
**a** Box plots of the original first-order mean values based on the stone type grouped by keV level. **b** Unsupervised clustering heatmaps comparing the radiomics signature of the different kidney stone types from 40 keV to 190 keV

At lower energy levels, particularly at 40 keV and to a lesser extent at 70 keV, there is a notable variation in the mean Hounsfield Unit (HU) values of the kidney stones based on their type. For instance, brushite and uric acid stones exhibit significantly higher HU values compared to struvite and xanthine stones, with brushite averaging around 1200 HU and xanthine around 250 HU at 40 keV. However, at higher energy levels, no significant differences in the Original_Firstorder_Mean parameter are observed between the stone types (Fig. 4a).

The displayed heat maps of the extracted radiomics parameters showed no significant differences in the various monoenergetic levels investigated in unsupervised clustering (Fig. 4b).

**Fig. 5:**
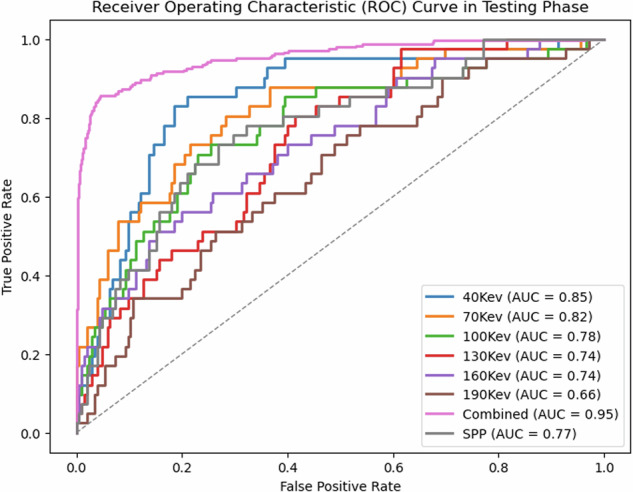
Results of the RF Classifiers for the different monoenergetic reconstructions, the SPP reconstruction as well as the combined model

Random forest classifiers were performed with the radiomics parameters of the individual monoenergetic reconstructions, the SPP reconstruction and in a combined, spectral-driven approach with all extracted parameters from all reconstructed monoenergetic levels. The combined approach performed best by far, with an AUC of 0.95 (40 keV performed second best with an AUC of 0.85). The other monoenergetic levels as well as the SPP reconstruction performed with a lower accuracy (70 keV performed with an AUC of 0.82, 100 keV with an AUC of 0.78, 130 keV with an AUC of 0.74, 160 keV with an AUC of 0.74, 190 keV with an AUC of 0.66 and SPP with an AUC of 0.77) (Fig. 5). The test accuracy for differentiating the different stone types was 0.81 with a train accuracy of 0.99 (Table 2).

**Table 2:** Train/test accuracy of the random forest classifier of the combined, spectral-driven radiomics model approach

Training data				
Stone type	Precision	Recall	F1-score	Support
Brushite	0.99	0.99	0.99	80
Carbonate apatite	1.00	1.00	1.00	84
Cystine	1.00	1.00	1.00	143
Struvite	0.97	0.96	0.96	71
Uric acid	0.98	0.98	0.98	130
Xanthine	1.00	1.00	1.00	59
Accuracy			0.99	567
Macro average	0.99	0.99	0.99	567
Weighted avg	0.99	0.99	0.99	567

Test data				
Stone type	Precision	Recall	F1-score	Support
Brushite	0.81	0.76	0.79	34
Carbonate apatite	0.89	0.69	0.78	36
Cystine	0.83	0.85	0.84	61
Struvite	0.75	0.77	0.76	31
Uric acid	0.77	0.88	0.82	56
Xanthine	0.92	0.88	0.90	25
Accuracy			0.81	243
Macro average	0.83	0.81	0.81	243
Weighted avg	0.82	0.81	0.81	243

**Fig. 6:**
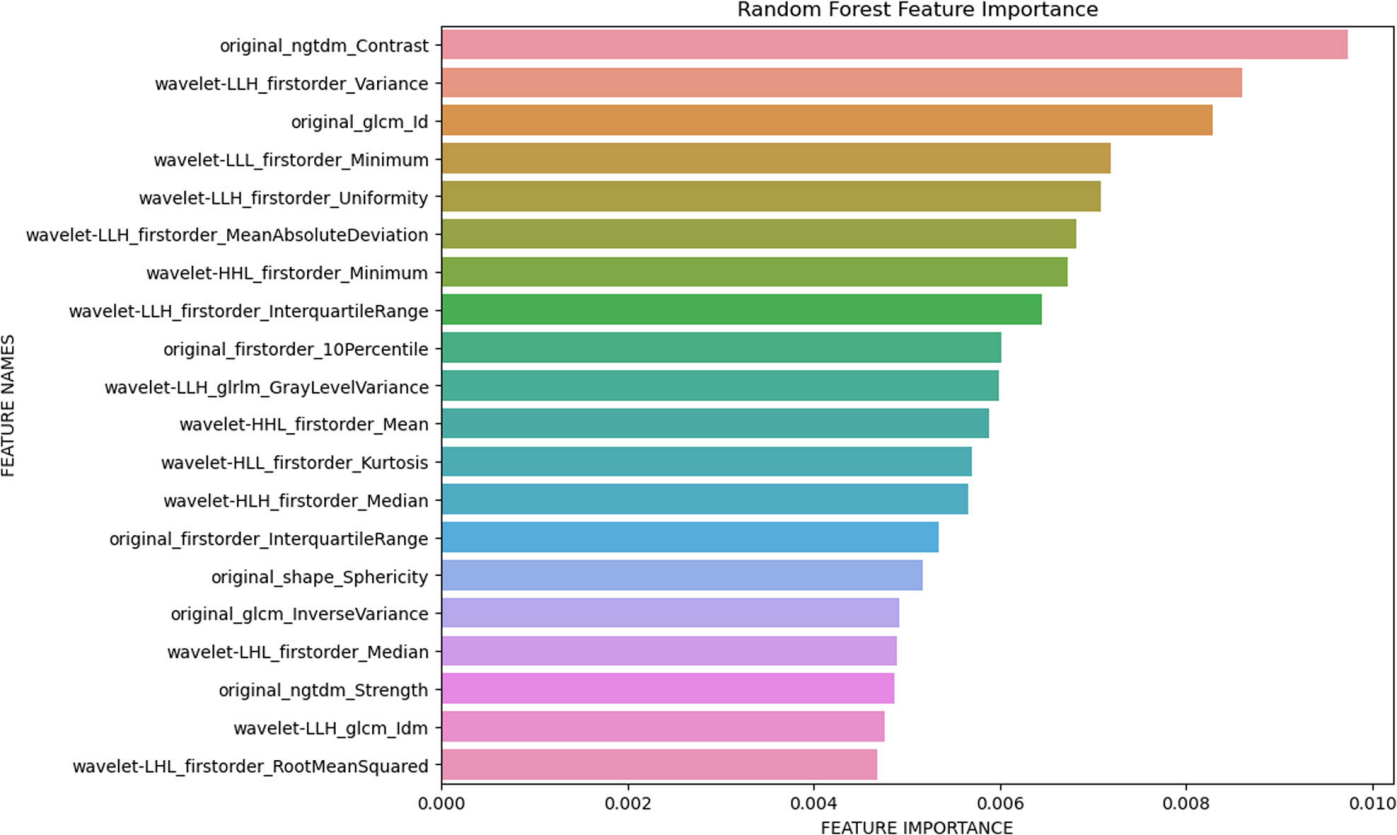
Results of the feature importance analysis

In the feature importance analysis, several radiomics parameters were identified as key for distinguishing between different kidney stone types. Notably, original_NGDTM_Strength, wavelet-LLH_firstorder_Variance, and original_glcm_Id emerged as important parameters for this differentiation (Fig. 6).

## Discussion

The new PCCT technology provides a novel outlook for improving diagnostic accuracy in medical imaging. In this study, we demonstrated the potential of spectral-driven radiomics analysis based on different monoenergetic reconstruction of PCCT scans to differentiate the different kidney stone types by employing a random forest classifier in an ex vivo setting. With that algorithm. it is possible to differentiate between six different types of kidney stones with a test accuracy of 0.81 and an AUC of 0.95. This high accuracy could be achieved by combining the multispectral information (the classifiers trained with radiomics parameters of single monoenergetic levels showed significantly lower accuracies). In addition, our analyses identified several radiomics features important for kidney stone differentiation. Here, the Original_ngtdm_Strength feature is particularly noteworthy, as it is known to hold substantial interpretative value regarding the domain of texture analysis, as it facilitates a nuanced understanding of the surface characteristics of the examined object [35].

There are several studies on the automated detection of kidney stones. However, they used different target variables and modalities requiring closer elucidation. Leng et al demonstrated a sensitivity of 73.1% in differentiating between uric acid (UA) stones and non-uric acid (Non-UA) stones based on two consecutive spatially registered low- and high-energy scans acquired on a conventional CT system presenting a first differentiation model categorizing into super-ordinated groups [36]. Moreover, using dual-energy computed tomography (DECT) instead of conventional CT, Primak et al demonstrated an accuracy of 100% in medium and large phantom sizes and an accuracy of 93% in very large phantom sizes. In addition, they simulated the in vivo situation by acquiring the images of the calculi placed inside porcine kidneys [37]. However, this study also differentiated between UA and non-UA stones and did not precisely differentiate non-UA stones. In addition, the total number of calculi examined was rather limited (*n* = 40). As a high dataset is essential for representative training, we analyzed a larger sample size to ensure a higher generalizability of the result.

The evolution of CT technology, particularly the advent of DECT, has introduced new opportunities for enhanced diagnostic precision and non-invasive stone characterization. Accurate measurement of stone size is critical for determining appropriate intervention, yet conventional CT has demonstrated some variability. Studies have shown that multiplanar reformation with bone window settings offers the most accurate size measurements, closely matching manual caliper-based measurements of kidney stones. Axial images, especially those with varying slice thicknesses, tend to underestimate stone size, potentially impacting clinical decisions. Importantly, the influence of reconstruction algorithms, such as model-based iterative reconstruction (MBIR), was found to enhance measurement accuracy, particularly when using sharp kernels [14].

In parallel, DECT presents a significant advancement in stone assessment by enabling non-invasive differentiation of stone compositions. DECT utilizes two distinct energy levels to provide material-specific attenuation values, allowing for differentiation between uric acid and non-uric acid stones, which is pivotal for guiding treatment strategies. Virtual non-contrast images derived from DECT have been shown to provide comparable detection rates to conventional CT at low iodine-induced attenuation levels (≤ 400 HU). However, at higher attenuation levels and lower radiation doses, stone detection rates decrease, particularly for smaller stones, underscoring the importance of protocol optimization [38].

Another significant area of advancement is the role of radiation dose reduction in CT imaging. PCCT has demonstrated the potential to significantly lower radiation doses while maintaining superior image quality. Compared to conventional CT, PCCT showed improved signal-to-noise ratio and overall image quality, allowing for the reliable detection of kidney stones with less radiation exposure. The potential for further reductions in dose, particularly with ultra-low-dose protocols, makes PCCT a promising tool for future clinical applications [39].

Moreover, a classification into distinct stone types (xanthine, brushite, carbonate apatite, cystine, struvite, uric acid, and whewellite stones) has a significantly higher diagnostic impact, which could ultimately allow early and targeted therapy of patients [40]. We have thus reasonably extended previously described investigations with our results and, in addition, used the most advanced techniques of radiologic CT imaging. An attempt at a closer differentiation was performed by Kaviani et al investigating the differentiability of calcium oxalate stones and urate stones. Using the radiomics parameters of single-energy CT, they demonstrated an AUC of 0.78 and a sensitivity of 0.79 for the differentiation of calcium oxalate and urate stones [41]. The potential of radiomics to differentiate stone types was likewise verified in our study. We were able to show a comparable sensitivity, however, the multispectral PCCT scans could distinguish not only between calcium and urate stones, but between six different stone types, respectively.

In addition to the techniques we applied in this study, deep-learning-based methods have shown promise in improving image quality for the detection of urolithiasis, especially in low-dose CT settings. Deep-learning-based image denoising techniques (DLID) have demonstrated significant reductions in image noise and improvements in signal-to-noise and contrast-to-noise ratios compared to conventional methods such as filtered-back projection and hybrid iterative reconstruction. Studies, such as the one by Terzis et al, have shown that DLID achieves comparable diagnostic accuracy for urinary stone detection to MBIR while significantly reducing image noise [42].

Integrating deep-learning models into future applications for stone detection may offer advantages in terms of both diagnostic performance and dose reduction, particularly in settings requiring ultra-low-dose CT. These methods could complement photon-counting CT, potentially enhancing the differentiation of kidney stone types while minimizing radiation exposure.

There are some limitations to this study that need to be addressed. With a total of 135 different kidney stones stored in phantom containers, the number of samples is slightly limited based on the availability of suitable stones complying with preanalytical criteria. However, compared with similar studies already published, the number of samples is in a comparable range. Additionally, the kidney stones were not scanned in vivo, but ex vivo, stored in a phantom container. Here, there would most likely be dissonance with in vivo scans regarding the extracted radiomics parameters. However, since the phantom enclosures consist of meat and water, a reasonably comparable scanning environment was created. Thus, we targeted this limitation when establishing the study design to simulate a representative ex vivo milieu as feasible.

Moreover, a major problem in the clinical establishment of radiomics-based approaches is the well-known problem of radiomics feature instability, in which many different factors such as CT device, scan parameters, as well as even the way in which segmentation was performed need to be considered. Prior studies have already shown the high feature stability of scans acquired with the photon-counting CT [26].

In addition, due to the massive quantity of data, it is often difficult for readers to reconstruct radiomics evaluations and these evaluations often suffer from transparency deficits. For this reason, we have focused on methodological aspects in this work to ensure reproducibility.

It is crucial to acknowledge that the methodologies deployed in this study leveraging PCCT are not confined to renal stone characterization alone. Their potential extends into diverse domains, such as distinguishing varied histological tumor subtypes or predicting mutations, akin to performing a virtual biopsy. For instance, Tharmaseelan et al demonstrated the capability of conventional CT-based radiomics features, in conjunction with machine learning, to discern the origin of liver metastases between pancreatic and colorectal cancers with an AUC of 0.87 and a sensitivity of 0.67 [43]. Given PCCT’s superior spatial resolution, enhanced texture uniformity, and improved stability of radiomics features, we anticipate that these attributes could contribute to achieving even greater sensitivities in PCCT-based studies in the future [44].

## Conclusion

In this work, we were able to differentiate six different types of uric acid and non-uric acid-containing kidney stones using multispectral radiomics analysis of PCCT scans with a test accuracy of 0.81 and an AUC of 0.95. We identified several radiomics parameters relevant to stone differentiation. It was shown that the six stone types differ, sometimes significantly, in terms of mean density in the low-energy reconstructions (especially 40 keV). This ex vivo workflow of this analysis could potentially lead to an optimization of treatment algorithms of patients with uro- or nephrolithiasis by the non-invasive differentiation of stone types due to the combination of innovative PCCT techniques and radiomics-based quantitative image analysis and leads the way for further in vivo studies.

## Abbreviations

CT: Computed tomography
DECT: Dual energy computed tomography
DLID: Deep-learning-based image denoising
glcm: Grey level co-occurrence matrix
gldm: Grey level dependence matrix
glrlm: Grey level run length matrix
glszm: Grey level size zone matrix
HU: Hounsfield Unit
ngtdm: Neighboring Grey Tone Difference Matrix
Non-UA: Non-urinary acid
PCCT: Photon-counting computed tomography
SPP: Spectral post-processing
UA: Urinary acid
